# A novel gene signature related to focal adhesions for distinguishing and predicting the prognosis of lung squamous cell carcinoma

**DOI:** 10.3389/fmed.2023.1284490

**Published:** 2024-01-08

**Authors:** Gang Hui, Yuancai Xie, Li Niu, Jixian Liu

**Affiliations:** ^1^Department of Thoracic Surgery, Peking University Shenzhen Hospital, Shenzhen, China; ^2^Shenzhen Cheerland Biotechnology Co., Ltd., Southern University of Science and Technology, Shenzhen, China; ^3^CheerLand Clinical Laboratory Co., Ltd., Peking University Medical Industrial Park, Beijing, China

**Keywords:** lung squamous cell carcinoma (LUSC), prognosis, focal adhesion, focal adhesion-related signature, novel gene signature, biomarkers

## Abstract

**Background:**

Lung squamous cell carcinoma (LUSC) is a devastating and difficult-to-treat type of lung cancer, and the prognosis of LUSC is the worst. The functional roles of focal adhesion-related genes were explored in LUSC based on data from The Cancer Genome Atlas (TCGA).

**Methods:**

RNA sequencing data and clinical characteristics of LUSC patients in TCGA-LUSC were obtained from the TCGA database. Through systematic analysis, we screened the prognostic genes and determined the focal adhesion-related pathways closely associated with LUSC.

**Results:**

We identified 444 prognostic genes and focal adhesion-related pathways intimately associated with LUSC. According to the focal adhesion-related genes, TCGA-LUSC patients were well divided into two groups: the low-risk group (G1) and the high-risk group (G2). A differential expression analysis identified 44 differentially expressed genes (DEGs) upregulated in the low-risk G1 group and 379 DEGs upregulated in the high-risk G2 group. The upregulated DEGs in the G1 group were primarily related to tyrosine metabolism, steroid hormone biosynthesis, retinol metabolism, platinum drug resistance, pentose and glucuronate interconversions, and metabolism of xenobiotics by cytochrome P450, while the downregulated DEGs in the G1 group were primarily related to ECM-receptor interaction, focal adhesion, proteoglycans in cancer, small cell lung cancer, cytokine-cytokine receptor interaction, and TGF-beta signaling pathway. The immune activity of the G1 group was lower than that of the G2 group, and the half-maximal inhibitory concentration (IC50) of five chemotherapy drugs (i.e., gemcitabine, methotrexate, vinorelbine, paclitaxel, and cisplatin) was significantly different between the G1 and G2 groups. Furthermore, a 10-gene prognostic model was constructed to predict the prognosis for LUSC patients: *ITGA3*, *VAV2*, *FLNC*, *FLT4*, *HGF*, *MYL2*, *ITGB1*, *PDGFRA*, *CCND2*, and *PPP1CB*.

**Conclusion:**

The status of focal adhesion-related genes has a close relationship with tumor classification and immunity in LUSC patients. A novel focal adhesion-related signature had good prognostic and predictive performance for LUSC. Our findings may provide new insight into the diagnosis and treatment of LUSC.

## Introduction

The morbidity and mortality of lung cancer rank first among malignant tumors in the world, among which non-small cell lung cancer (NSCLC) accounts for 80–85% (1). NSCLC encompasses a group of cancer types, such as adenocarcinoma (LUAD), squamous cell cancer (LUSC), and large cell cancers (2). Among them, LUSC accounts for approximately 30% of NSCLC (1, 2). LUSC, a devastating and difficult-to-treat type of lung cancer, is currently treated with radiotherapy and chemotherapy, with few targeted drugs (3). LUSC patients not only experience great pain and suffering but also have a poor prognosis (4). The 5-year survival rate of metastatic LUSC is less than 5% (2–4). In the past 20 years, little progress has been made in the field of LUSC treatment, especially in the first-line treatment, resulting in a serious unmet medical need, and more first-line treatment options are urgently needed in the advanced group.

At present, the vast majority of NSCLC are in the advanced stage of relapse or metastases when they are clinically diagnosed, and chemotherapy is the main treatment (1). However, as the standard first-line treatment, platinum-based drugs combined with chemotherapy have entered a plateau. In recent years, research on molecular targeted therapy has made people see the hope of crossing this platform, and it is now a hot spot and trend in lung cancer treatment (5).

Focal adhesions are the main connection between cells and the extracellular matrix and participate in cell movement and signal transduction (6). Among them, targeting molecules related to the focal adhesion pathway has become a potential therapeutic strategy for the treatment of tumors (7). For example, focal adhesion kinase (FAK) is a non-receptor tyrosine kinase that participates in tumor proliferation, migration, invasion, and angiogenesis by integrating signals from extracellular pressure, cell adhesion, etc. (8). Many FAK-related signal transduction pathways have become molecular markers for tumor diagnosis and important targets for malignant tumor treatment (8). TAE226 is an ATP-competitive tyrosine kinase inhibitor targeting FAK, which can effectively block the FAK signaling pathway (9). With the deepening of research, it was found that TAE226 has obvious anti-proliferation, migration, and invasion effects on oral squamous cell carcinoma (9). However, the role of the focal adhesion signaling pathway in LUSC remains to be further studied.

## Materials and methods

### Data sets of the TCGA-LUSC cohort

RNA sequencing data and clinical characteristics of lung squamous cell carcinoma patients in TCGA-LUSC were obtained from The Cancer Genome Atlas (TCGA) database.^1^

### Identification of prognostic genes in LUSC

Univariate Cox analyses were performed to find out the prognostic-related genes in LUSC patients, and the top 20 prognostic genes were presented with a *P*-value of <0.05. The Kyoto Encyclopedia of Genes and Genomes (KEGG) pathway enrichment analysis was performed to identify signaling pathways of prognostic genes in LUSC with the “ClusterProfiler” R package. We further explored the interactions of prognostic genes in LUSC to construct a protein-protein interaction (PPI) network using the STRING website^2^ using R cluster profilter, and cytoscape was performed to present the PPI network.

### Identification of tumor subtypes based on the focal adhesion-related pathway in LUSC

To explore the relationship between the expression of focal adhesion-related genes and LUSC subtypes, consistent cluster analyses were conducted on the TCGA-LUSC cohort using the R package ConsensusClusterPlus (version 1.54.0). The clustering variable (k) was set from 2 to 6. The heatmap was generated by the pheatmap R package (version 1.0.12). The overall survival (OS) time was compared between the different subgroups through Kaplan-Meier analysis.

### Identification of focal adhesion-related DEGs in LUSC

The differentially expressed genes (DEGs) between different LUSC clusters were identified using “DEseq2” R package. A *P*-value of <0.05 and |log 2-fold change| > 1 were regarded as the cutoff criterion. Furthermore, volcano plots were generated by the “ggplot2” R package to visualize the DEGs. The heatmap of DEGs was generated by the pheatmap R package (version 1.0.12). Gene Ontology (GO) and KEGG analyses were conducted by applying the “ClusterProfiler” package.

### Immune activity between two focal adhesion-related clusters in LUSC

Based on focal adhesion-related genes, immune activity was evaluated by CIBERSORT in “immunoeconomics”. Eight immune checkpoint-related genes were used to compare the immune activity of two focal adhesion-related clusters: *CD274*, *PDCD1*, *PDCD1LG2*, *CTLA4*, *LAG3*, *HAVCR2*, *TIGIT*, and *SIGLEC15*. The heatmap and boxplot were generated by the pheatmap package and the R package ggplot2, respectively. The Wilcox test was used to compare the immune cell infiltration and immune pathway activation between the two groups. A *P*-value of <0.05 was considered statistically significant.

### Drug susceptibility of the two clusters

Drug sensitivity analysis was used to explore the difference between two focal adhesion-related clusters in LUSC using the pRRophetic algorithm. The chemotherapy response of each patient was evaluated by the available pharmacogenomics database.^3^ The prediction process was performed by the “pRRophetic” R software package. The ridge regression was used to estimate the half-maximal inhibitory concentration (IC50). A *P*-value of <0.05 was considered statistically significant.

### Development of the focal adhesion-related gene prognostic model

To evaluate the prognostic value of the focal adhesion-related genes, Cox regression analysis was further employed to construct the prognostic model in the TCGA-LUSC cohort with the “glmnet”R package. The variables were non-zero coefficients, and the lambda condition was decided by the minimum criteria. The risk score was as follows: Risk score = sum (expression level of each gene × corresponding coefficient). The TCGA-LUSC patients were divided into low- and high-risk subgroups based on the median risk score. The OS time was compared between the different subgroups through Kaplan-Meier analysis using the “survival” R package. The hazard ratios (HRs) with 95% confidence intervals (CIs) were calculated using the Cox proportional hazards analysis.

## Results

### Identification of the prognostic genes in LUSC

**FIGURE 1:**
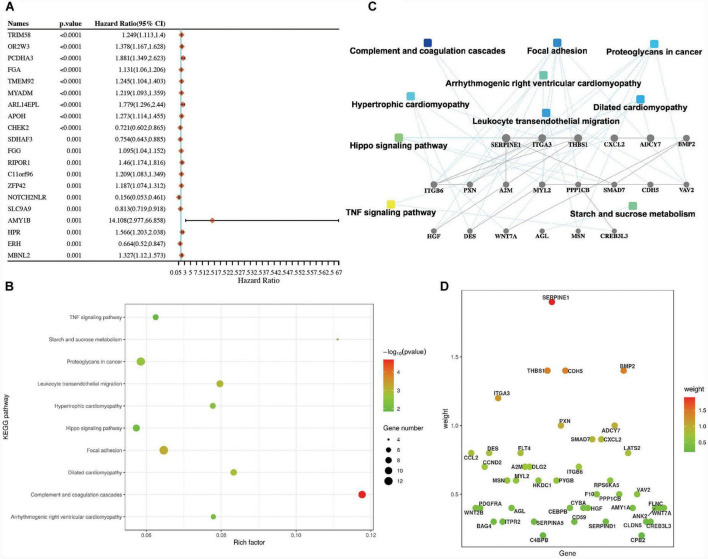
Identification of the prognostic genes in lung squamous cell carcinoma (LUSC). **(A)** Top 20 prognostic genes in TCGA-LUSC patients according to the *P*-value. **(B)** The KEGG enrichment pathways of the prognostic genes in LUSC. **(C)** PPI networks of the prognostic genes in LUSC. **(D)** The prognostic genes of the KEGG pathway in LUSC.

To explore the roles of the prognostic genes in LUSC, univariate Cox analyses were performed to find out the prognostic-related genes in TCGA-LUSC patients. There were 444 prognostic genes determined by the univariate Cox analysis in LUSC. The top 20 prognostic genes are shown in Figure 1A and Supplementary Table 1. Moreover, we further explored the roles of the 444 prognostic genes using the KEGG enrichment analysis. The KEGG enrichment results suggested that these pathways, including the TNF signaling pathway, focal adhesion, proteoglycans in cancer, and leukocyte transendothelial migration, might play important roles in LUSC (Figure 1B and Supplementary Table 1).

The PPI networks of these prognostic-related genes were also confirmed. These pathways, which included the TNF signaling pathway, focal adhesion, proteoglycans in cancer, and complement and coagulation cascades, were mainly involved in LUSC (Figure 1C and Supplementary Table 1). The genes related to the KEGG pathway were also identified in Figure 1D, such as focal adhesion (including *ITGA3*, *VAV2*, *FLNC*, *FLT4*, *HGF*, *ITGB6*, *THBS1*, *MYL2*, *ITGB1*, *CCND2*, *PPP1CB*, *PXN*, and *PDGFRA*) and complement and coagulation cascades (including *SERPINE1*, *SERPIND1*, *SERPINA5*, *F10*, *CD59*, *CPB2*, and *A2M*).

### LUSC subtypes based on the focal adhesion-related genes

Considering focal adhesions are closely related to the development of tumors (6, 7), the role of the focal adhesion signaling pathway in LUSC remains to be further studied. The correlations between gene expression and immune score were analyzed using Spearman.

**FIGURE 2:**
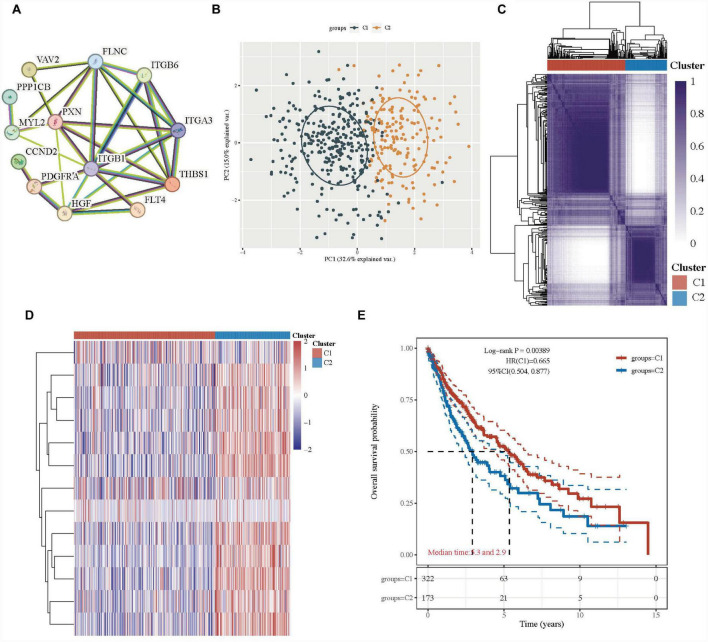
Lung squamous cell carcinoma subtypes based on the focal adhesion-related genes. **(A)** PPI networks of the prognostic focal adhesion genes in the focal adhesion signaling pathway. **(B)** The principal component analysis of two clusters in TCGA-LUSC patients. **(C)** Consensus clustering matrix of two clusters in LUSC. **(D)** Heatmap of two clusters in LUSC. **(E)** Kaplan–Meier OS curves for the two clusters.

First, we analyzed the connections between focal adhesion-related genes (*ITGA3*, *VAV2*, *FLNC*, *FLT4*, *HGF*, *ITGB6*, *THBS1*, *MYL2*, *ITGB1*, *CCND2*, *PPP1CB*, *PXN*, and *PDGFRA*) in the focal adhesion signaling pathway, and these genes widely interacted (Figure 2A and Supplementary Table 2), indicating they might play important roles in LUSC. The 13 focal adhesion-related genes were next applied to stratify TCGA-LUSC patients into different subtypes using the consensus clustering analysis.

When the clustering variable (k) was 2, the TCGA-LUSC patients could be well stratified into two clusters with a consistency cluster analysis and principal component analysis (PCA) (Figures 2B–C). The gene expression profiles of 13 focal adhesion-related genes were well separated in a heatmap between the two groups in TCGA-LUSC patients (Figure 2D). Interestingly, there was a statistically significant tendency for better overall survival time in cluster 1 than those in group 2 (HR: 0.665, 95% CI: 0.504–0.877, *P* = 0.00389; Figure 2E and Supplementary Table 2).

### Identification of the underlying mechanism between two groups in LUSC

**FIGURE 3:**
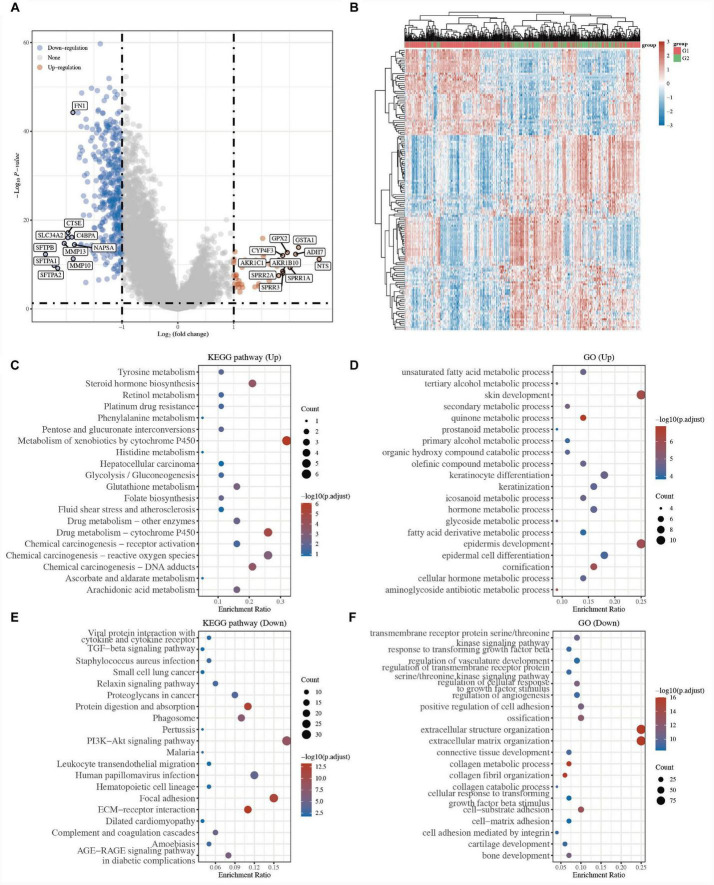
Identification of the underlying mechanism between two clusters in LUSC. **(A)** The volcano plots of DEGs in G1 LUSC samples compared to G2. Red refers to the upregulated genes and blue refers to the downregulated genes. **(B)** Heatmap of the top 50 differentially expressed genes. The enriched KEGG pathways of the upregulated **(C)** and downregulated DEGs **(E)**. The enriched biological processes of the upregulated **(D)** and downregulated DEGs **(F)**.

To understand the different mechanisms of the two groups, under the cutoff of *P* < 0.05 and |log 2-fold change| > 1, 423 DEGs, including 44 upregulated genes and 379 downregulated genes, were identified between the two groups in the volcano plot (G1 vs. G2; Figure 3A and Supplementary Table 3). The gene expression of the top 50 DEGs showed a different tendency between the two clusters in the heatmap (Figure 3B and Supplementary Table 3).

To better explore the biological roles of 423 DEGs, GO and KEGG enrichment analyses were performed. The results of KEGG analysis revealed that 44 upregulated genes were mainly related to metabolism and biosynthesis, for example, tyrosine metabolism, steroid hormone biosynthesis, retinol metabolism, platinum drug resistance, pentose and glucuronate interconversions, metabolism of xenobiotics by cytochrome P450, and drug metabolism-cytochrome P450 (Figure 3C and Supplementary Table 3), while 379 downregulated genes were mainly related to hallmarks of cancer, for example, ECM-receptor interaction, focal adhesion, proteoglycans in cancer, small cell lung cancer, cytokine-cytokine receptor interaction, and TGF-beta signaling pathway (Figure 3E and Supplementary Table 3). Similarly, the GO for BP results showed that the upregulated genes were primarily associated with unsaturated fatty acid metabolic processes, skin development, epidermis development, tertiary alcohol metabolic processes, glycoside metabolic processes (Figure 3D and Supplementary Table 3), while the downregulated genes were primarily associated with extracellular matrix organization, collagen fibril organization, collagen metabolic process, cell-substrate adhesion, positive regulation of cell adhesion, and regulation of cellular response to growth factor stimulus (Figure 3F and Supplementary Table 3). Many studies have proven that focal adhesion, proteoglycans in cancer, and cytokine-cytokine receptor interaction are tumor markers (10, 11).

The above results indicated that the tumor cells of the G2 LUSC subtypes might have stronger tumor migration and proliferation ability.

### Immune activity between two focal adhesion-related groups in LUSC

**FIGURE 4:**
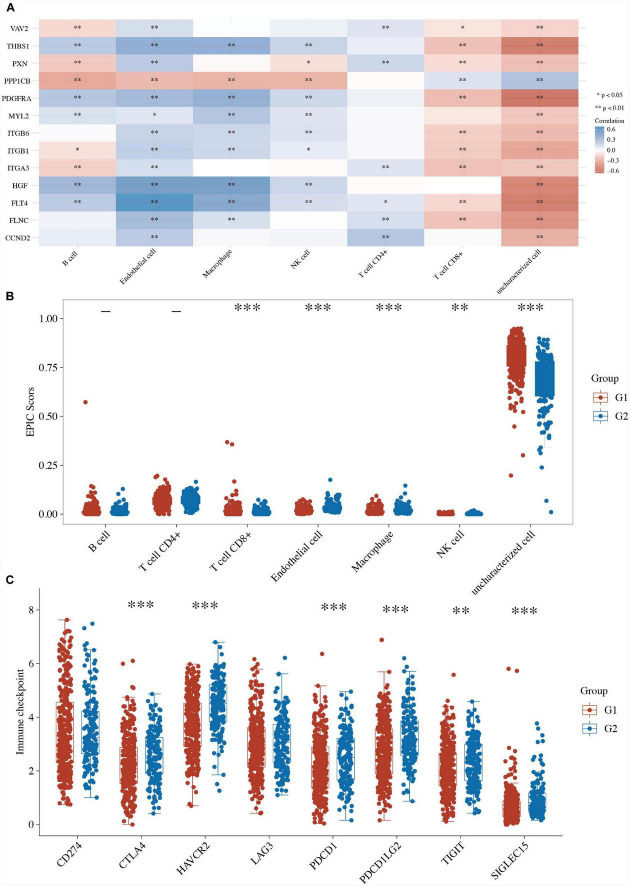
Immune activity between two focal adhesion-related clusters in LUSC. **(A)** The correlations between the expression of focal adhesion genes and immune score. **(B)** Comparison of the enrichment scores of 7 types of immune cells between two focal adhesion-related clusters in LUSC. **(C)** Comparison of the gene expression of the immune checkpoint inhibitors between two clusters in LUSC. *P < 0.05; **P < 0.01; ***P < 0.001.

Many studies have proven that focal adhesion is closely related to immune activity in many cancers (6–8). First, the correlations between the expression of focal adhesion genes and immune score were analyzed using Spearman. The focal adhesion-related genes (*ITGA3*, *VAV2*, *FLNC*, *FLT4*, *HGF*, *ITGB6*, *THBS1*, *MYL2*, *ITGB1*, *CCND2*, *PPP1CB*, *PXN*, and *PDGFRA*) were closely correlated with many immune cells (Figure 4A and Supplementary Table 4). We further compared the immune activity between two focal adhesion-related clusters in LUSC patients. The boxplots revealed that the immune cells, including the T cells CD8^+^, macrophages, NK cells, and dendritic cells, of the C1 LUSC samples were clearly different from those of the G2 (Figure 4B and Supplementary Table 4).

Furthermore, the boxplots also revealed that 6 of the 8 ICI-related genes (i.e., *CTLA4*, *HAVCR2*, *PDCD1*, *PDCD1LG2*, *TIGIT*, and *SIGLEC15*) were lower in the G1 LUSC sample than the G2 patients (Figure 4C and Supplementary Table 4). These results suggested a close relationship between focal adhesion and immune activity.

### Drug susceptibility of the two groups in LUSC

**FIGURE 5:**
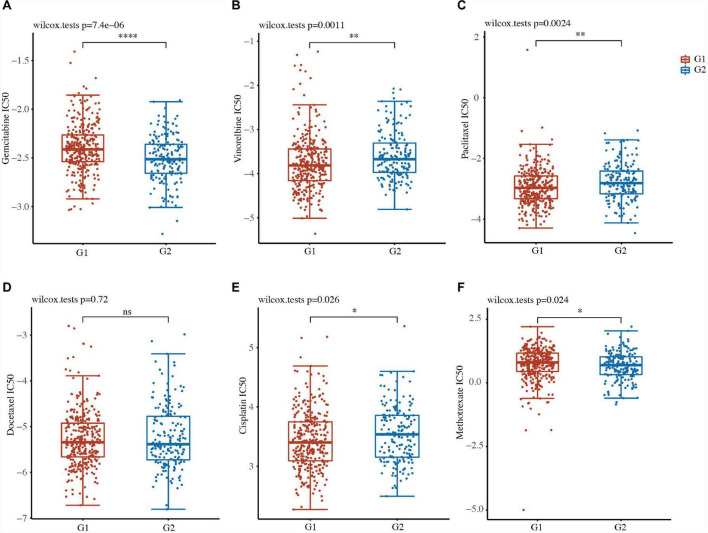
Drug susceptibility of the two clusters in LUSC. **(A–F)** IC50 values of gemcitabine **(A)**, vinorelbine **(B)**, paclitaxel **(C)**, docetaxel **(D)**, cisplatin **(E)**, and methotrexate **(F)** of the two clusters in LUSC. *P < 0.05; **P < 0.01; ***P < 0.001; ****P < 0.0001.

We further evaluated the drug sensitivity of the two clusters in LUSC using the pRRophytic algorithm. Six chemotherapy drugs, namely gemcitabine, vinorelbine, paclitaxel, docetaxel, cisplatin, and methotrexate, were selected to perform the drug sensitivity analysis between the two clusters in LUSC. We found that the status of the two clusters was closely associated with the IC50 scores of gemcitabine, vinorelbine, paclitaxel, cisplatin, and methotrexate in LUSC (Figures 5A–F and Supplementary Table 5). The IC50 values of gemcitabine and methotrexate were lower in the high-risk G2 LUSC patients than in the G1 patients (Figures 5A–F and Supplementary Table 5). The IC50 values of vinorelbine, paclitaxel, and cisplatin were higher in the high-risk G2 LUSC patients than in the G1 patients (Figures 5A–F and Supplementary Table 5). These results suggested focal adhesion-related classification might represent a good predictor of response to chemotherapy.

### Construction of a prognostic model based on focal adhesion-related genes

**FIGURE 6:**
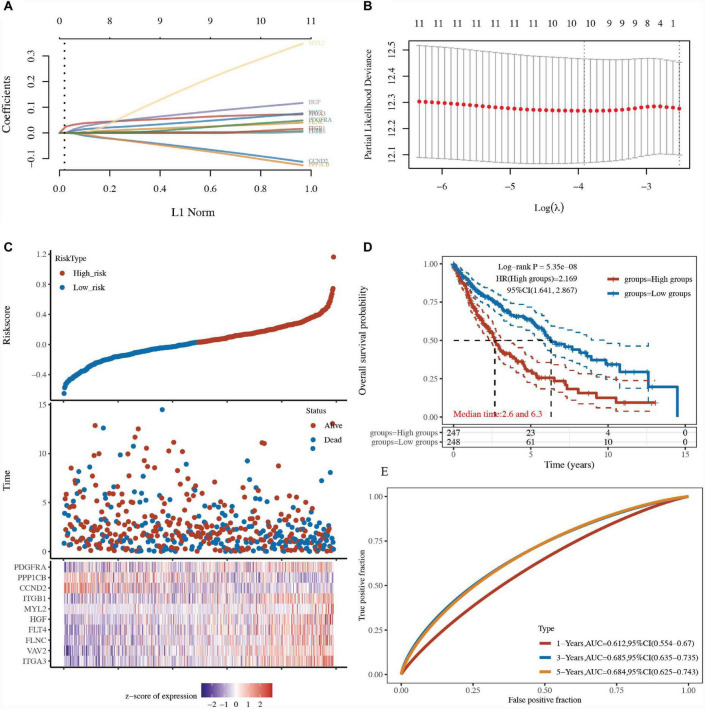
Construction of a prognostic model based on focal adhesion-related genes. **(A)** LASSO regression of 13 focal adhesion-related genes. **(B)** Cross-validation of the LASSO regression. **(C)** Patients divided by the median risk score in the TCGA-LUSC cohort. The levels of 13 focal adhesion-related genes in the high- and low-risk groups were presented in red and blue, respectively. **(D)** Survival analysis between the high-risk and low-risk groups in the TCGA-LUSC cohort. **(E)** AUC curves indicated the accuracy of the prognostic model in the TCGA-LUSC cohort.

LASSO and Cox regression analyses were further performed to select the 13 focal adhesion-related genes in LUSC. A 10-gene signature was developed based on the optimum λ value. The formula used to calculate the risk score was as follows: risk score = ITGA3 expression × 0.0669 + VAV2 expression × 0.0542 + FLNC expression × 0.0284 + FLT4 expression × 0.0034 + HGF expression × 0.0919 + MYL2 expression × 0.246 + ITGB1 expression × 0.0017 + PDGFRA expression × 0.0297 + CCND2 expression × (−0.0758) + PPP1CB expression × (−0.0847) (Figures 6A, B and Supplementary Table 6). Based on this gene signature, TCGA-LUSC patients were divided into low-risk and high-risk groups (Figure 6C). The overall survival analysis revealed that patients with a low-risk score had better survival than patients with a high-risk score (HR: 2.169, 95% CI: 1.641–2.867, *P* = 5.35e–08; Figure 6D and Supplementary Table 6). A receiver operating characteristic (ROC) curve was used to test the accuracy of the prognostic model. The area under the curve (AUC) was 0.6126 for 1 year, 0.685 for 3 years, and 0.684 for 5 years (Figure 6E and Supplementary Table 6), suggesting that this model had a fine prognostic effect.

### Comparison of tumor stemness between LUSC subtypes

**FIGURE 7:**
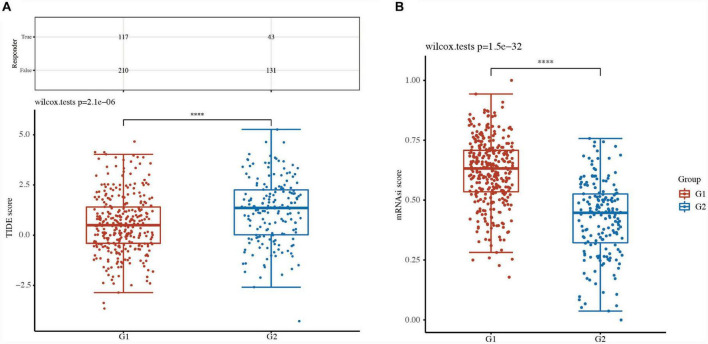
Comparison of tumor stemness between LUSC subtypes. **(A)** Tumor immune dysfunction and exclusion (TIDE) scores and **(B)** cancer stem cell score between two clusters in LUSC. ****P < 0.0001.

A high TIDE score is associated with a poor response to immune checkpoint blockade (ICB) treatment and a short survival period after receiving ICB treatment. The TIDE score of G2 TCGA-LUSC patients was higher than that of G1 patients, indicating a poor response to ICB therapy and poor overall survival (Figure 7A).

Cancer stem cells (CSCs) are major factors contributing to tumor development, relapse, and metastasis and are responsible for chemotherapy resistance and cancer recurrence. The CSC scores of G2 TCGA-LUSC patients were significantly different from those of G1 patients (Figure 7B). These results suggested that focal adhesion-related classification might represent a good predictor of response to ICB therapies.

## Discussion

In this study, 444 prognostic genes were obtained, and focal adhesion-related pathways were intimately associated with LUSC using the KEGG enrichment analysis. TCGA-LUSC patients were well divided into low-risk and high-risk groups based on the 13 focal adhesion-related genes. The upregulated DEGs in the G1 group were primarily related to tyrosine metabolism, steroid hormone biosynthesis, retinol metabolism, platinum drug resistance, pentose and glucuronate interconversions, and metabolism of xenobiotics by cytochrome P450, while the downregulated DEGs in the G1 group were primarily related to ECM-receptor interaction, focal adhesion, proteoglycans in cancer, small cell lung cancer, cytokine-cytokine receptor interaction, and TGF-beta signaling pathway. The immune activity of the G1 group was lower than that of the G2 group, and the IC50 scores of five chemotherapy drugs (i.e., gemcitabine, methotrexate, vinorelbine, paclitaxel, and cisplatin) were significantly different between the G1 and G2 groups. Furthermore, a 10-gene prognostic model was constructed to predict the prognosis for LUSC patients: *ITGA3*, *VAV2*, *FLNC*, *FLT4*, *HGF*, *ITGB1*, *MYL2*, *ITGB1*, *PDGFRA*, *CCND2*, and *PPP1CB*.

We summarized the similarities and differences between these previous articles and our manuscript. In these articles, all the subjects had lung squamous cell carcinoma, which was similar to our study. However, the difference was in the methods of selecting genes. In Zhang et al.’s research, basement membrane-related genes were used to construct the prognosis model (12). Zhai et al. identified LUSC subtypes based on inflammation-related genes, while we identified low-risk C1 subtypes and high-risk C2 subtypes based on 13 focal adhesion-related genes and constructed the prognosis model based on the screening of 10 focal adhesion-related genes (13). The apoptotic protein poly ADP ribose polymerase (PARP) is a key modulator of cancer cell growth and survival (14). Excitingly, PARP7 is a therapeutic target that, when inhibited, induces both cancer cell-autonomous and immune stimulatory effects through enhanced IFN signaling (15). Oral administration of RBN-2397, a small-molecule inhibitor of PARP7, leads to complete tumor regression in a lung cancer xenograft and triggers tumor-specific adaptive immune memory in an immunocompetent mouse model of cancer (15).

Through systematic analysis, we aimed to investigate the role of focal adhesion in LUSC. Focal adhesions have been found to mainly connect cells and the extracellular matrix and have been regarded as a great potential therapeutic strategy for the treatment of tumors (8). For example, TAE226, an inhibitor of focal adhesion kinase (FAK), inhibits cell proliferation, migration, and invasion in oral squamous cell carcinoma (9). Moreover, defactinib (VS-6063), a FAK and proline-rich tyrosine kinase 2 (PYK2) dual inhibitor, is currently undergoing multiple clinical trials in NSCLC and mesothelioma (16). However, the role of the focal adhesion signaling pathway and LUSC requires further study.

TCGA-LUSC patients were well stratified into two groups (low risk and high risk) based on focal adhesion-related genes. A functional enrichment analysis of high-risk G1 and low-risk G2 of TCGA-LUSC patients further demonstrated that the focal adhesion-related pathway and the cancer-related pathway (proteoglycans in cancer, small cell lung cancer, and cytokine-cytokine receptor interaction) might be associated with LUSC. The high-risk G1 subgroup had lower levels of immune activity than the low-risk G2 subgroup. The IC scores of five chemotherapy drugs (i.e., gemcitabine, methotrexate, vinorelbine, paclitaxel, and cisplatin) were significantly different between the G1 and G2 subgroups. These results thus suggested that tumor classification for LUSC was well separated into two subgroups according to the focal adhesion-related genes.

The expression of focal adhesion-related genes was positively correlated with prognosis in LUSC patients, and 10 focal adhesion-related genes were determined using the prognostic model for LUSC patients: *ITGA3*, *VAV2*, *FLNC*, *FLT4*, *HGF*, *MYL2*, *ITGB1*, *PDGFRA*, *CCND2*, and *PPP1CB*. Integrin alpha-3 (*ITGA3*) regulates cancer cell migration and invasion in head and neck cancer (17). Guanine nucleotide exchange factor (*VAV2*) was associated with lung cancer cell adhesion by regulating focal adhesion kinase activity (18). Filamin-C (*FLNC*) was associated with the risk of glioblastoma multiforme (19). Vascular endothelial growth factor receptor 3 (*FLT4*) enhances cervical cancer migration and invasion (20). Hepatocyte growth factor (*HGF*) increased cisplatin resistance through downregulation of AIF in lung cancer (21). Integrin beta-1 (*ITGB1*) is related to the tumorigenesis and progression of gastric cancer (22). Inhibiting platelet-derived growth factor receptor alpha (*PDGFRA*) enhanced radioiodine sensitivity in thyroid cancer (23). G1/S-specific cyclin-D2 (*CCND2*) was associated with cell proliferation and apoptosis in breast cancer (24).

Nevertheless, this study also has some limitations of its own. The specific role of focal adhesions on LUSC should be further elucidated via *in vitro* and *in vivo* verifications. In future, we could focus on the exploration of the specific mechanism of focal adhesion genes in LUSC progression, which may provide new strategies for the treatment of LUSC.

## Conclusion

The status of focal adhesion-related genes has a close relationship with tumor classification and immunity in LUSC patients. A novel focal adhesion-related signature had good prognostic and predictive performance for LUSC. Our findings may provide new insight into the diagnosis and treatment of LUSC.

## Data availability statement

The original contributions presented in this study are included in this article/Supplementary material, further inquiries can be directed to the corresponding author.

## Author contributions

GH: Conceptualization, Data curation, Methodology, Formal analysis, Investigation, Writing – original draft, Writing – review and editing. YX: Data curation, Conceptualization, Investigation, Writing – review and editing. LN: Data curation, Writing – review and editing. JL: Funding acquisition, Conceptualization, Project administration, Resources, Supervision, Writing – original draft, Writing – review and editing.

## Footnotes

1
https://portal.gdc.cancer.gov/repository

2
https://string-db.org/

3
https://www.cancerrxgene.org/
